# Effects of Systemic or Local Administration of Zoledronate on Implant Osseointegration: A Preclinical Meta-Analysis

**DOI:** 10.1155/2019/9541485

**Published:** 2019-09-22

**Authors:** Yao He, Wei Bao, Xiang-Dong Wu, Wei Huang, Hong Chen, Zhengyun Li

**Affiliations:** ^1^Department of Orthopedics, Banan People's Hospital of Chongqing, Chongqing, China; ^2^Department of Orthopedics, The First Affiliated Hospital of Chongqing Medical University, Chongqing, China; ^3^Department of Orthopedic Surgery, Peking Union Medical College Hospital, Chinese Academy of Medical Sciences & Peking Union Medical, Beijing, China

## Abstract

**Objective:**

This study aims to investigate the effect of systemically administrated zoledronate on bone-implant fixation in animal models.

**Methods:**

We searched MEDLINE, Embase, and EBSCO for studies that explore the role of systemic or local zoledronate delivery in implant osseointegration in animal models. The Review Manager software was used to analyze selected studies by using the weighted mean difference random-effects model. Analytical data are mainly about bone ingrowth, such as bone-to-implant contact (BIC), bone volume/total volume (BV/TV), and bone area.

**Results:**

Twenty studies were selected from 182 publications. The mean quality score was 18/20 for all of the 20 studies (*κ* = 0.9). Despite differences in protocols, these studies showed consistent improvement of implant osseointegration with zoledronate administration. In addition, the osteoporotic animal model, systemic or local administration, sufficient drug dosage, and sample follow-up time were correlated with improved outcomes.

**Conclusion:**

Systematic administration of zoledronate could improve the osseointegration of orthopedic implant in animal models. Results of this meta-analysis should be interpreted cautiously because of the inherent differences between preclinical and clinical subjects. For the local administration, there is a similar trend as well, but the results need to be confirmed and complemented with further analyses.

## 1. Introduction

Bone ingrowth into a prosthetic implant is crucial for the longevity of uncemented total hip arthroplasty (THA). Rapid and sound bone ingrowth can increase implant stability and improve long-term bone-implant fixation. In addition, adequate tissue ingrowth may protect the bone-implant interface against wear particle-induced osteolysis, which further decreases the risk of aseptic loosening [1]. Therefore, how to improve bone-implant fixation is always a topic of great interest for joint surgeons.

Zoledronate (ZOL) is a new-generation intravenous bisphosphonate (BP) with the greatest affinity and longest retention for bone mineral, and it has been largely utilized in the treatment of osteoporosis and metastatic bone disease. It has a well-documented profile of possible side effects, such as initial influenza-like illness which has been documented with the first infusion of BPs. Renal failure has been noted in patients with cancer after repetitive high-dose infusions, and an association between BPs and osteonecrosis of the jaw after tooth extraction has been recorded as well [2, 3]. ZOL is traditionally believed to be an antiresorptive agent; however, recent animal studies suggested that it could stimulate bone formation and improve implant mechanical fixation [4, 5]. Meanwhile, there are clinical studies revealing that ZOL is associated with decreased early implant migration and reduced peri-implant bone loss [6, 7]. Nevertheless, apart from these findings, because of small sample sizes and diverse study protocols of previous researches, the exact cellular and molecular mechanisms governing the improved bone content, structure, and strength, induced by the systemically or locally administered ZOL on implant osseointegration, have no consensus. Thus, a preclinical meta-analysis was performed to investigate the effect of systemically or local administrated ZOL on bone-implant fixation in animal models and to guide the design of evidence-based, large-scale preclinical or clinical trials.

## 2. Materials and Methods

### 2.1. Inclusion and Exclusion Criteria

Inclusion criteria for the literature were as follows: (1) original animal studies, (2) studies that aimed to explore the role of ZOL delivery in implant osseointegration, (3) studies that included a control group, which received placebo or no drug, and (4) studies with the outcomes that included pertinent information regarding bone ingrowth, such as bone-to-implant contact (BIC), bone volume/total volume (BV/TV), and bone area. Exclusion criteria were as follows: (1) clinical studies, (2) implants embedded in the mandible or maxilla, and (3) presence of other confounding factors, such as wear debris in a local environment.

### 2.2. Search Strategy

Literature, which was published before September 30, 2018, was searched by using the electronic databases MEDLINE, Embase, and EBSCO. No language restriction was applied. The adopted search keywords were “implant AND (bisphosphonates OR zoledronate) AND osseointegration AND arthroplasty.” Titles and abstracts of studies that fulfill the eligibility criteria were screened by the authors and checked for agreement. Finally, the reference lists of all full-text papers, which were identified pertinent to the study, were reviewed for any unidentified studies.

### 2.3. Study Selection

Two authors (Yao He and Zhengyun Li) independently applied the search strategy to select references from the aforementioned databases. The titles and abstracts were reviewed independently. When in doubt, the full-text articles were retrieved for further examination. These two authors independently assessed each full report to evaluate fulfillment of the inclusion criteria, and corresponding authors were contacted for more information and clarification regarding their data, if necessary. Any disagreement was discussed with the senior author, and when consensus could not be established, that study was excluded.

### 2.4. Quality Assessment

The methodological quality of included studies was assessed independently by two authors (Yao He and Xiang-Dong Wu) according to the ARRIVE guidelines which included title, abstract, background, objectives, ethical statement, study design, experimental procedures, experimental animals, housing and husbandry, sample size, allocation of animals to experimental groups, experimental outcomes, statistical methods, baseline data, numbers analyzed, outcomes and estimation, adverse events, interpretation/scientific implications, generalizability/translation, and funding. Each study was given a quality score out of a possible total of 20 points. Any disagreement was resolved by the senior author (Yao He).

### 2.5. Data Extraction

A data extraction form was designed and agreed by the authors, and a pilot test of five articles was performed to ensure their consistency. Initially, two authors (Yao He and Xiang-Dong Wu) independently extracted the data, which were later reviewed jointly to produce the agreed accurate data. Disagreements were resolved by consensus or consultation with the senior author. The extracted data included study design, animal species, implantation site, implant characteristics (material, shape, and coating), ZOL route and dosage, follow-up time, and outcome measurements (BIC, BV/TV, and bone area). In all studies, BIC was calculated as the length percentage of the direct bone-implant interface to the total implant surface, BV/TV was defined as the percentage of mineralized bone volume to total bone tissue volume in the peri-implant region, and bone area was evaluated as the percentage of bone tissue area to the total area of the bone and implant.

### 2.6. Statistical Analysis

Review Manager (RevMan version 5.0, The Cochrane Collaboration in 2008) was used to analyze the included studies. The primary outcome was the BIC between treatment and control groups. From a clinical point of view, the authors (Yao He and Xiang-Dong Wu) performed subgroup analyses according to the animal model (osteoporotic or normal), animal species, and drug dosage and frequency, as well as follow-up time. In case of multiple treatment groups next to a control group within one trial, the animal number in the control group was divided equally by the number of treatment groups. For each arm in a particular study, continuous data were expressed as means and standard deviations (SDs), and dichotomous data were expressed as proportions or risks. For continuous outcomes, we calculated the mean differences (MDs) with 95% confidence interval (CI). For dichotomous outcomes, we estimated the relative risks' 95% CI. Statistical heterogeneity was assessed by using the value of *I*^2^ and the result of the chi-square test. An *I*^2^ value >50% suggests statistical heterogeneity, which prompts a random-effects modeling estimate. Otherwise, a fixed-effects approach was used. A *P* value <0.05 was determined as statistically significant.

## 3. Results

### 3.1. Included Studies

**Figure 1:**
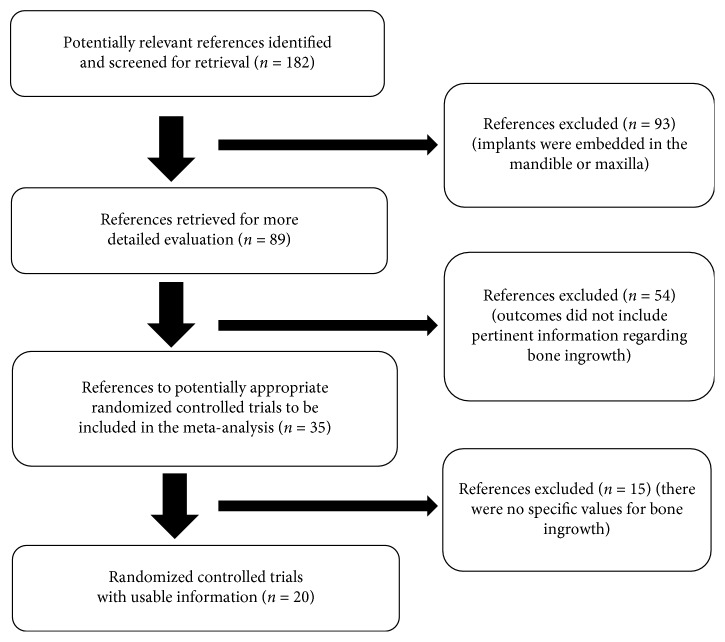
Flow of studies through the review.

A total of 182 articles were searched from multiple electronic databases. After screening their titles and available abstracts, 20 satisfied the eligibility criteria and were included in the meta-analysis [8–27] (Figure 1).

### 3.2. Characteristics of Enrolled Studies

The sample size ranged from 10 to 64. In twelve studies, rats were used as animal models; in other seven studies, rabbits were utilized as animal models; and in the last one study, dogs were used as animal models. The follow-up time ranged from 10 days to 1 year. The tail vertebra was used as the implantation site in one study; in another four studies, the femoral condyle was operated on; the remaining 15 studies all selected the proximal tibia as the surgical site. Out of 20 studies, one used the tantalum as the implant, another used the calcium phosphate bone cement, and the remaining eighteen studies used the titanium implant (Table 1).

### 3.3. Methodological Quality

The mean quality score was 18/20 for all of the 20 studies (Table 2) (*κ* = 0.9). Among all publications, eighteen (90%) reported the animal breeding condition, fourteen (70%) reported random allocation, no study reported blinded surgical implantation, and only one (5%) reported blinded outcome assessment. One study (5%) reported sample size calculation.

## 4. Systemic Administration

### 4.1. Bone-to-Implant Contact

**Figure 2:**
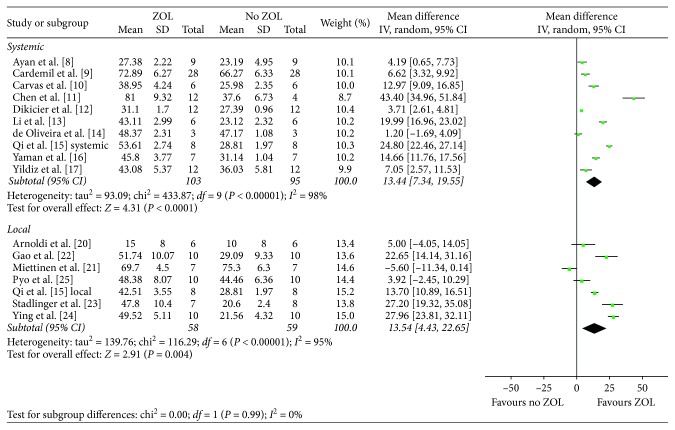
Forest plot of comparison for bone-to-implant contact between control and treatment groups.

Ten articles [8–17] with 193 animals reported BIC measurements with substantial heterogeneity between studies (*I*^2^ = 98%, *P* < 0.001); thus, the random-effects model was used to evaluate the results. A significant difference of BIC could be found between the treatment and control groups (MD, 13.44; 95% CI, 7.34–19.55; *P* < 0.0001) (Figure 2).

**Figure 3:**
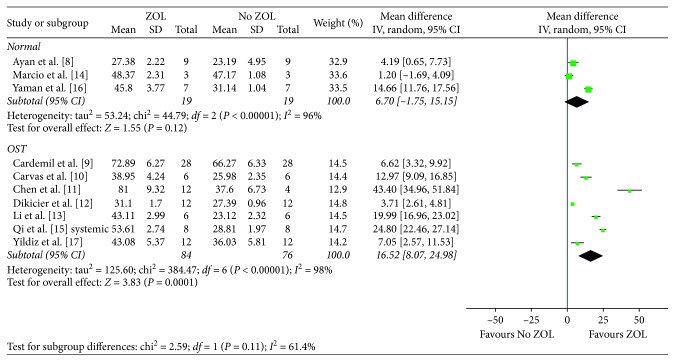
Subgroup analysis with regard to the animal model (osteoporotic or normal) in the systemic group. Bone-to-implant contact was significantly improved in osteoporotic animals.

Subgroup analyses were performed according to the animal model (osteoporotic *vs.* normal), animal species (rats *vs.* rabbits), ZOL dosage (>0.1 mg/kg *vs.* <0.1 mg/kg), administration frequency (single *vs.* multiple), and follow-up time (>8 weeks *vs.* <8 weeks). As shown in Figure 3, ZOL could significantly increase BIC in osteoporotic animals (mean difference, 16.52; 95% CI, 8.07–24.98; *P*=0.0001); however, this effect was not obvious in normal animals (MD, 6.70; 95% CI, −1.75 to 15.15; *P*=0.12). Based on animal species, similar effects of ZOL on BIC were observed for both rats (MD, 12.61; 95% CI, 5.50–19.72; *P*=0.0005) and rabbits (MD, 13.89; 95% CI, 5.80–21.98; *P*=0.0008).

**Figure 4:**
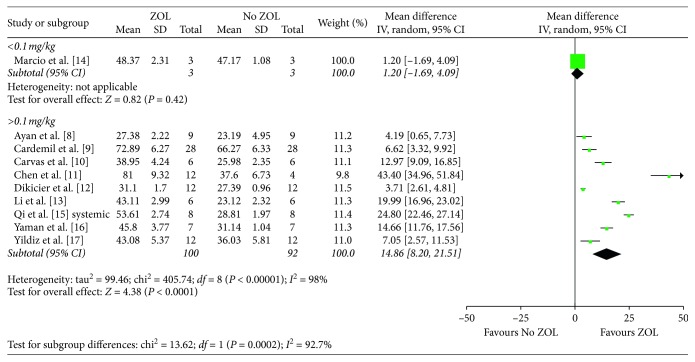
Subgroup analysis with regard to the animal model (<0.1 mg/kg or >0.1 mg/kg) in the systemic group.

Regarding the drug dosage, when ZOL dosage exceeded 0.1 mg/kg, BIC could be significantly improved compared with the control group (MD, 14.86; 95% CI, 8.20–21.51; *P* < 0.001) (Figure 4). On the contrary, administration frequency had no significant impact on BIC (test for subgroup difference: *P*=0.86). Finally, although positive influence of ZOL on BIC has been demonstrated in short follow-up time studies (<8 weeks; MD, 3.92; 95% CI, 0.69–7.14; *P*=0.02), this effect was much more significant in case of longer follow-up time (>8 weeks; MD, 17.65; 95% CI, 9.30–26.06; *P* < 0.001).

### 4.2. Bone Volume/Total Volume

**Figure 5:**
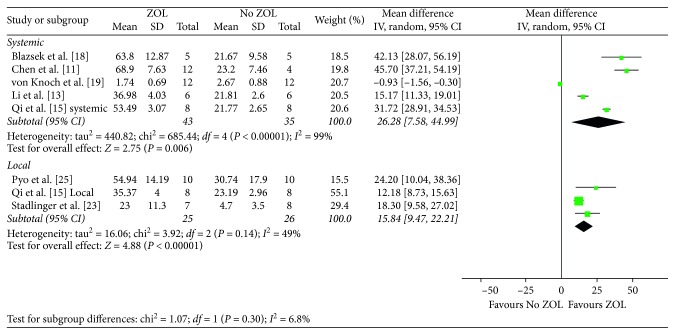
Forest plot of comparison for bone volume/total volume between control and treatment groups.

The pooled analysis of five experiments [11, 13, 15, 18, 19] showed a significant difference of BV/TV between ZOL-treated and control groups (MD, 26.28; 95% CI, 7.58–44.99; *P*=0.006) with heterogeneity (*I*^2^ = 99%, *P* < 0.001) (Figure 5).

Animal model (osteoporotic *vs.* normal) was initially used for subgroup analysis. The results showed that more effects of ZOL were seen improving BV/TV in osteoporotic animals (MD, 22.28; 95% CI, 11.98–32.58; *P*=0.0004). With regard to animal species, ZOL significantly increased BV/TV in rats (MD, 18.60; 95% CI, 4.59–32.60; *P*=0.009) and rabbits (MD, 8.00; 95% CI, 3.47–12.53; *P*=0.0005). Finally, similar effects of ZOL on bone area were observed for follow-up time <8 weeks (MD, 13.19; 95% CI, 1.22–25.17; *P*=0.03) and >8 weeks (MD, 20.12; 95% CI, 6.81–33.43; *P*=0.003). ZOL has a significant effect on BV/TV if ZOL dosage was more than 0.1 mg/kg (MD, 32.85; 95% CI, 20.15 to 45.55; *P* < 0.001) or if ZOL was given in multiple doses (MD, 28.52; 95% CI, 14.66 to 42.39; *P* < 0.001).

Animal model (osteoporotic *vs.* normal) was initially used for subgroup analysis. The results showed that ZOL could only improve BV/TV in osteoporotic animals (MD, 30.43; 95% CI, 16.05–44.80; *P* < 0.001), but not in normal ones (MD, 20.00; 95% CI, −22.18 to 62.19; *P*=0.35). With regard to animal species, ZOL significantly increased BV/TV in rats (MD, 44.75; 95% CI, 37.48–52.01; *P* < 0.001); however, this effect was not evidenced in rabbits (MD, 15.29; 95% CI, −7.01 to 37.59; *P*=0.18). Finally, ZOL has an effect on BV/TV if ZOL dosage was more than 0.1 mg/kg (MD, 32.85; 95% CI, 20.15–45.55; *P* < 0.001) and if ZOL was given in multiple doses (MD, 28.52; 95% CI, 14.66–42.39; *P* < 0.001).

### 4.3. Bone Area

**Figure 6:**
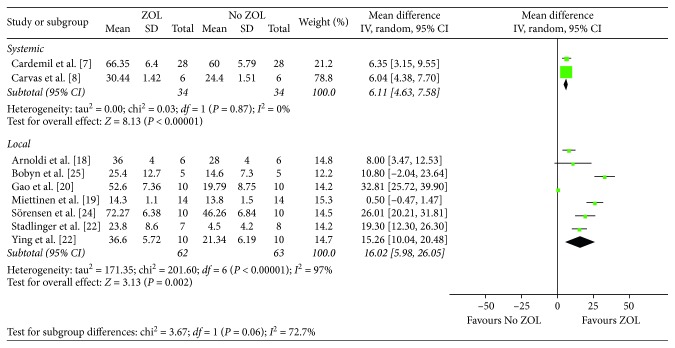
Forest plot of comparison for bone area between control and treatment groups.

Only two studies [9, 10] with 68 animals reported bone area, and no heterogeneity was observed (*I*^2^ = 0, *P*=0.87) (Figure 6).

## 5. Local Administration

### 5.1. Bone-to-Implant Contact

Seven articles [20–25] with 117 animals reported BIC measurements with substantial heterogeneity between studies (*I*^2^ = 95%, *P* < 0.001); thus, the random-effects model was used to evaluate the results. A significant difference of BIC could be found between the treatment and control groups (MD, 13.54; 95% CI, 4.43–22.65; *P*=0.004) (Figure 2).

**Figure 7:**
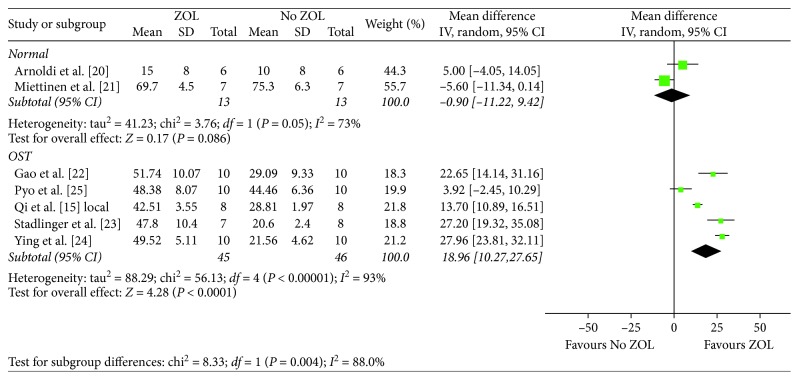
Subgroup analysis with regard to the animal model (osteoporotic or normal) in the local group.

Subgroup analyses were performed according to the animal model (osteoporotic *vs.* normal), animal species (rats *vs.* rabbits *vs.* dogs), and follow-up time (>8 weeks *vs.* <8 weeks). As shown in Figure 7, ZOL with local administration could significantly increase BIC in osteoporotic animals (MD, 18.96; 95% CI, 10.27–27.65; *P* < 0.0001); but in normal animals, there was no effect at all (MD, −0.9; 95% CI, −11.22 to 9.42; *P*=0.86).

Based on animal species, similar effects of ZOL on BIC were observed for rats (MD, 15.16; 95% CI, 0.66–29.66; *P*=0.04) and rabbits (MD, 10.46; 95% CI, 2.21–18.70; *P*=0.01). On the contrary, only the longer follow-up time has a increased effect on BIC (MD, 17.05; 95% CI, 7.21–26.89; *P* < 0.0007).

### 5.2. Bone Volume/Total Volume

Only three studies [15, 23, 25] with 51 animals reported bone volume/total volume with heterogeneity between studies (*I*^2^ = 49%, *P*=0.14) (Figure 4).

### 5.3. Bone Area

A total of seven articles [21–24, 26, 27] with 125 animals reported bone area showing a significant difference between ZOL-treated and no ZOL groups (MD, 16.02; 95% CI, 5.98–26.05; *P*=0.002) with heterogeneity (*I*^2^ = 97%, *P* < 0.001) (Figure 6).

## 6. Discussion

THA is an effective technique owing to its ability to reduce pain, correct deformity, and improve function. However, its longevity is always an unsolved issue. According to previous reports, the most common reason of implant failure is aseptic loosening, which is caused by implant micromotion, prosthesis-related stress shielding, disuse osteoporosis, and wear-debris-induced osteolysis [6]. Thus, a simple, low-cost, and readily available method for improving implant fixation and decreasing periprosthetic bone loss is considerably important. Over the recent years, some animal study data proposed that ZOL might increase peri-implant bone stock and improve biological implant fixation, whereas other studies have denied this effect [14]. By pooling the currently available animal study data, the present meta-analysis provides evidence-based information about the positive effects of ZOL on implant osseointegration. In addition, our results indicated that the animal model, drug dosage, and follow-up time might influence study outcomes, which suggests possible reasons for the diversity of previous studies and gives insights into the design of future research.

Although bisphosphonates are well-known osteoclast inhibitors, they could reduce bone resorption by inhibiting and promoting apoptosis of osteoclasts [28, 29]. Several in vitro studies have demonstrated that they could also stimulate osteoblast function [30, 31]. However, according to our results, ZOL could only significantly improve implant osseointegration in osteoporotic animals but not in normal ones, which indicates that this effect was mediated mainly by decreasing the abnormal bone turnover rate rather than directly stimulating bone formation. Meanwhile, caution should be taken when interpreting this result. In the experimental animals, osteoporosis was acquired mainly by ovariectomy and was the only systemic condition. Nevertheless, in clinical settings, osteoporotic patients are usually old aged and sometimes diabetic. It has already been reported that aging and chronic hyperglycemia would lead to accumulation of advanced glycation end products (AGEs), which could negatively influence bone metabolism, and thus, the effect of ZOL may be less promising in clinical settings than in laboratories [32, 33].

In view of the widespread use of bisphosphonates and the increase in bisphosphonate-related cases of osteonecrosis of the jaw, some studies have shown that osteonecrosis with dental implants may be a side effect of treatment with BP. The incidence of bisphosphonate-related osteonecrosis of the jaws is accelerated at the end of or during BP treatment. Serra et al. [34] suggested the avoidance of dental implant procedures in patients that have been receiving intravenous BPs. A recent review indicates that one hundred percent of the studies related to combined use of BPs have shown cases of osteonecrosis [35]. Others authors [36–41] suggested that bisphosphonate exposure and implant placement do not affect implant success and do not result in osteonecrosis. However, the duration of their follow-up was short. Najeeb et al. [42] believe that these results should be confirmed by more in-depth research before the dental implant can be used in the clinic. This is also the reason that one of the exclusion criteria is the implants embedded in the mandible or maxilla in our study.

Rats are the most commonly used animal model for osteoporosis studies because the ovariectomized rat exhibits most of the characteristics of human postmenopausal osteoporosis. However, the lack of intracortical remodeling process in this animal compromises the physiologic investigation of the cortical bone. By contrast, rabbits do have some inherent advantages as the osteoporosis animal model. For example, they achieve skeletal maturity shortly after reaching complete sexual development and show significant intracortical remodeling [43]. Thus, some researchers prefer rabbits as their ideal model. With respect to our results, studies with rats or rabbits have achieved similar yet slightly different outcomes, indicating that different animal models may influence implant osseointegration characteristics. However, because of the limited number of included studies, drawing the final conclusion now is too early.

The dosage and frequency of ZOL delivery varied among the included studies, and the best medication administration protocol remains unclear. According to the present study, administration frequency does not exert much influence as long as drug dosage exceeds 0.1 mg/kg. This information is quite important because concerns about the safety of long-term bisphosphonate usage are always present. If single and multiple administrations have similar osseointegration-improving effects, long-term usage would be unnecessary, thus avoiding the risk of complications, such as osteonecrosis of the jaw or stress fracture [44].

This meta-analysis has several limitations. Firstly, because of the small number of included studies and the limited animal sample sizes, conclusions from this meta-analysis should be interpreted cautiously and should be substantiated by larger studies. Secondly, because of the diverse study characteristics, animal populations, and treatment protocols, significant heterogeneity existed among the included studies. Nevertheless, because the main focus of preclinical meta-analysis is to generate hypotheses, the existence of heterogeneity is quite rational and could provide insight into the design of future clinical trials [45].

## 7. Conclusions

In conclusion, current animal studies demonstrate that both systemic and local administration of ZOL could improve the osseointegration of the orthopedic implant in animal models. An appropriate animal model (osteoporotic), sufficient drug dosage (exceeding 0.1 mg/kg, only in the method of systemic administration), and enough follow-up time (more than eight weeks) are crucial influencing factors, which should be given particular attention in future animal or clinical studies. Nonetheless, caution should be taken when interpreting the results of this meta-analysis because of inherent differences between preclinical and clinical subjects.

## Figures and Tables

**Table 1:** Characteristics of enrolled studies.

Studies	Animals	Group		ZOL dosage and route, or concentration	Implant			FU time
		Number	Size		Site	Type	Coating	
Ayan et al. [8]	Male rabbits	1: control	9	0.1 mg/kg IV	Tibia	Titanium screw	No	4 weeks
		2: ZOL		Single dose				

Cardemil et al. [9]	Female rats	1: control (OVX)	28	0.1 mg/kg IV	Tibia	Titanium screw	No	4 weeks
		2: ZOL		Single dose				

Carvas et al. [10]	Male rabbits	1: control (GC)	6	0.1 mg/kg IV	Tibia	Titanium screw	No	18 weeks
		2: ZOL		Single dose				

Chen et al. [11]	Female rats	1: control (OVX)	12	0.1 mg/kg IV	Tibia	Titanium rod	HA	12 weeks
		2: ALN		Single dose				
		3: SR						
		4: ZOL						

Dikicier et al. [12]	Female rats	1: control (OVX)	12	0.04 mg/kg IV	Tibia	Titanium screw	No	8 weeks
		2: ZOL		6 doses				

Li et al. [13]	Female rabbits	1: control (OVX)	6	0.1 mg/kg SC	Tibia	Titanium screw	HA	8 weeks
		2: ZOL		3 doses				

de Oliveira et al. [14]	Male rats	1: control	3	0.0075 mg/kg SC	Tibia	Titanium	No	4 weeks
		2: ZOL		3 doses				

Qi et al. [15]	Female rabbits	1: control (OVX)	8	0.1 mg/kg SC	Tibia	Titanium screw	HA	12 weeks
		2: local ZOL		4 doses				
		3: systemic ZOL						
		4: local and systemic ZOL						

Yaman et al. [16]	Male rats	1: control	7	0.1 mg/kg IV	Tibia	Titanium screw	HA	12 weeks
		2: ZOL		3 doses				

Yildiz et al. [17]	Female rabbits	1: control (OVX)	12	0.1 mg/kg IV	Tibia	Titanium screw	Resorbable blast media	8 weeks
		2: ZOL		Single dose				

Blazsek et al. [18]	Female rats	1: control	5	0.6 mg/kg IP	Tail vertebra	Titanium screw	No	6 weeks
		2: ZOL		3 doses				

von Knoch et al. [19]	Rabbits	1: control	12	0.015 mg/kg IV	Femur	Titanium cylinder	Fiber metal mesh	12 weeks
		2: ZOL		Single dose				

Arnoldi et al. [20]	Rabbits	1: control	6	1 *μ*g/ml	Femur	Titanium screw	No	10 days
		2: ZOL						

Miettinen et al. [21]	Male rats	1: control	7	20 *μ*g/ml	Femur	Titanium	No	4 weeks
		2: ZOL						

Gao et al. [22]	Female rats	1: control (OVX)	10	1 mg/ml	Tibia	Titanium	HA	12 weeks
		2: ZOL						

Stadlinger et al. [23]	Female rats	1: control (OVX)	8	8.5 *μ*g/implant	Tibia	Titanium	No	4 weeks
		2: ZOL						

Ying et al. [24]	Female rats	1: control (OVX)	10	30 *μ*g/implant	Tibia	Titanium cylinder	No	12 weeks
		2: ZOL						

Pyo et al. [25]	Female rats	1: control (OVX)	10	8 *μ*g/ml	Tibia	Titanium screw	No	8 weeks
		2: ZOL		80 *μ*g/ml				
				800 *μ*g/ml				

Sörensen et al. [26]	Rats	1: control	10	50 *μ*g/implant	Tibia	Calcium phosphate bone cement	No	3 weeks
		2: ZOL						

Bobyn et al. [27]	Dogs	1: control	5	0.2 mg/ml	Femur	Tantalum	HA	1 year
		2: ZOL						

ZOL: zoledronate; FU: follow-up; GC: glucocorticoid; HA: hydroxyapatite; ALN: alendronate; SR: strontium ranelate; SC: subcutaneous; IP: intraperitoneal.

**Table 2:** Quality assessment score of enrolled studies.

Studies	Quality score
Ayan et al. [8]	18
Cardemil et al. [9]	17
Carvas et al. [10]	16
Chen et al. [11]	19
Dikicier et al. [12]	17
Li et al. [13]	17
Marcio et al. [14]	19
Qi et al. [15]	17
Yaman et al. [16]	19
Yildiz et al. [17]	18
Blazsek et al. [18]	17
von Knoch et al. [19]	18
Arnoldi et al. [20]	18
Miettinen et al. [21]	18
Gao et al. [22]	19
Stadlinger et al. [23]	19
Ying et al. [24]	19
Pyo et al. [25]	19
Sörensen et al. [26]	18
Bobyn et al. [27]	17

## Additional Points

*Strengths and Limitations of This Study*. (1) Both systemic and local administration of ZOL could improve the osseointegration of the orthopedic implant in animal models. (2) The osteoporotic model could be more effective to improve the osseointegration of the orthopedic implant than the normal model. (3) Sufficient drug dosage (exceeding 0.1 mg/kg, only the method of systemic administration) and enough follow-up time (more than eight weeks) are crucial influencing factors. (4) This meta-analysis should be considered cautiously and should be substantiated by larger studies. (5) Results of this meta-analysis should be interpreted cautiously because of the inherent differences between preclinical and clinical subjects.

## References

[1] Bobyn J. D., Jacobs J. J., Tanzer M. (1995). The susceptibility of smooth implant surfaces to peri-implant fibrosis and migration of polyethylene wear debris. *Clinical Orthopaedics and Related Research*.

[2] Body J.-J., Diel I., Bell R. (2004). Profiling the safety and tolerability of bisphosphonates. *Seminars in Oncology*.

[3] Marx R. E. (2003). Pamidronate (Aredia) and zoledronate (Zometa) induced avascular necrosis of the jaws: a growing epidemic. *Journal of Oral and Maxillofacial Surgery*.

[4] von Knoch M., Wedemeyer C., Pingsmann A. (2005). The decrease of particle-induced osteolysis after a single dose of bisphosphonate. *Biomaterials*.

[5] Wise L. M., Waldman S. D., Kasra M. (2005). Effect of zoledronate on bone quality in the treatment of aseptic loosening of hip arthroplasty in the dog. *Calcified Tissue International*.

[6] Friedl G., Radl R., Stihsen C., Rehak P., Aigner R., Windhager R. (2009). The effect of a single infusion of zoledronic acid on early implant migration in total hip arthroplasty. *The Journal of Bone and Joint Surgery-American Volume*.

[7] Scott D. F., Woltz J. N., Smith R. R. (2013). Effect of zoledronic acid on reducing femoral bone mineral density loss following total hip arthrplasty: preliminary results of a prospective randomized trial. *The Journal of Arthroplasty*.

[8] Ayan M., Dolanmaz D., Mihmanlı A., Ayan A., Kürkçü M. (2012). The effect of systemically administrated zoledronic acid on the osseointegration of dental implants. *Oral Diseases*.

[9] Cardemil C., Omar O. M., Norlindh B., Wexell C. L., Thomsen P. (2013). The effects of a systemic single dose of zoledronic acid on post-implantation bone remodelling and inflammation in an ovariectomised rat model. *Biomaterials*.

[10] Carvas J. S. B., Pereira R. M. R., Caparbo V. F. (2010). A single dose of zoledronic acid reverses the deleterious effects of glucocorticoids on titanium implant osseointegration. *Osteoporosis International*.

[11] Chen B., Li Y., Yang X., Xu H., Xie D. (2013). Zoledronic acid enhances bone-implant osseointegration more than alendronate and strontium ranelate in ovariectomized rats. *Osteoporosis International*.

[12] Dikicier E., Karaçaylı Ü., Dikicier S., Günaydın Y. (2014). Effect of systemic administered zoledronic acid on osseointegration of a titanium implant in ovariectomized rats. *Journal of Cranio-Maxillofacial Surgery*.

[13] Li J.-P., Li P., Hu J. (2014). Early healing of hydroxyapatite-coated implants in grafted bone of zoledronic acid-treated osteoporotic rabbits. *Journal of Periodontology*.

[14] de Oliveira M. A., Asahi D. A., Silveira C. A. E., Lima L. A. P. A., Glick M., Gallottini M. (2015). The effects of zoledronic acid and dexamethasone on osseointegration of endosseous implants: histological and histomorphometrical evaluation in rats. *Clinical Oral Implants Research*.

[15] Qi M., Hu J., Li J. (2012). Effect of zoledronate acid treatment on osseointegration and fixation of implants in autologous iliac bone grafts in ovariectomized rabbits. *Bone*.

[16] Yaman F., Ağaçayak S., Atilgan S. (2012). Effects of systemic zoledronic acid administration on osseointegration of hydroxyapatite-coated and resorbable blast material surface implants in rabbit models. *The International Journal of Oral & Maxillofacial Implants*.

[17] Yildiz A., Esen E., Kürkçü M., Damlar I., Dağlioğlu K., Akova T. (2010). Effect of zoledronic acid on osseointegration of titanium implants: an experimental study in an ovariectomized rabbit model. *Journal of Oral and Maxillofacial Surgery*.

[18] Blazsek J., Dobó Nagy C., Blazsek I. (2009). Aminobisphosphonate stimulates bone regeneration and enforces consolidation of titanium implant into a new rat caudal vertebrae model. *Pathology & Oncology Research*.

[19] von Knoch F., Eckhardt C., Alabre C. I., Schneider E., Rubash H. E., Shanbhag A. S. (2007). Anabolic effects of bisphosphonates on peri-implant bone stock. *Biomaterials*.

[20] Arnoldi J., Alves A., Procter P. (2014). Early tissue responses to zoledronate, locally delivered by bone screw, into a compromised cancellousbone site: a pilot study. *BMC Musculoskeletal Disorders*.

[21] Miettinen S. S. A., Jaatinen J., Pelttari A. (2009). Effect of locally administered zoledronic acid on injury-induced intramembranous bone regeneration and osseointegration of a titanium implant in rats. *Journal of Orthopaedic Science*.

[22] Gao Y., Luo E., Hu J., Xue J., Zhu S., Li J. (2009). Effect of combined local treatment with zoledronic acid and basic fibroblast growth factor on implant fixation in ovariectomized rats. *Bone*.

[23] Stadlinger B., Korn P., Tödtmann N. (2013). Osseointegration of biochemically modified implants in an osteoporosis rodent model. *European Cells and Materials*.

[24] Ying G., Bo L., Yanjun J., Lina W., Binquan W. (2016). Effect of a local, one time, low-dose injection of zoledronic acid on titanium implant osseointegration in ovariectomized rats. *Archives of Medical Science*.

[25] Pyo S. W., Kim Y. M., Kim C. S., Lee I. S., Park J. U. (2014). Bone formation on biomimetic calcium phosphate–coated and zoledronate-immobilized titanium implants in osteoporotic rat tibiae. *The International Journal of Oral & Maxillofacial Implants*.

[26] Sörensen T. C., Arnoldi J., Procter P. (2013). Locally enhanced early bone formation of zoledronic acid incorporated into a bone cement plugin vivo. *Journal of Pharmacy and Pharmacology*.

[27] Bobyn J. D., McKenzie K., Karabasz D., Krygier J. J., Tanzer M. (2009). Locally delivered bisphosphonate for enhancement of bone formation and implant fixation. *The Journal of Bone and Joint Surgery-American Volume*.

[28] Hughes D. E., Wright K. R., Uy H. L. (1995). Bisphosphonates promote apoptosis in murine osteoclasts in vitro and in vivo. *Journal of Bone and Mineral Research*.

[29] Jobke B., Milovanovic P., Amling M., Busse B. (2014). Bisphosphonate-osteoclasts: changes in osteoclast morphology and function induced by antiresorptive nitrogen-containing bisphosphonate treatment in osteoporosis patients. *Bone*.

[30] von Knoch F., Jaquiery C., Kowalsky M. (2005). Effects of bisphosphonates on proliferation and osteoblast differentiation of human bone marrow stromal cells. *Biomaterials*.

[31] Im G.-I., Qureshi S. A., Kenney J., Rubash H. E., Shanbhag A. S. (2004). Osteoblast proliferation and maturation by bisphosphonates. *Biomaterials*.

[32] Yamagishi S.-I. (2011). Role of advanced glycation end products (AGEs) in osteoporosis in diabetes. *Current Drug Targets*.

[33] Yamagishi S., Nakamura K., Inoue H. (2005). Possible participation of advanced glycation end products in the pathogenesis of osteoporosis in diabetic patients. *Medical Hypotheses*.

[34] Serra M. P., Llorca C. S., Donat F. J. (2008). Oral implants in patients receiving bisphosphonates: a review and update. *Medicina Oral Patología Oral y Cirugia Bucal*.

[35] de-Freitas N. R., Lima L. B., de-Moura M. B., Veloso-Guedes C. C., Simamoto-Júnior P. C., de-Magalhães D. (2016). Bisphosphonate treatment and dental implants: a systematic review. *Medicina Oral Patología Oral y Cirugia Bucal*.

[36] Shabestari G. O., Shayesteh Y. S., Khojasteh A. (2010). Implant placement in patients with oral bisphosphonate therapy: a case series. *Clinical Implant Dentistry and Related Research*.

[37] Fugazzotto P. A., Lightfoot W. S., Jaffin R., Kumar A. (2007). Implant placement with or without simultaneous tooth extraction in patients taking oral bisphosphonates: postoperative healing, early follow-up, and the incidence of complications in two private practices. *Journal of Periodontology*.

[38] Memon S., Weltman R. L., Katancik J. A. (2012). Oral bisphosphonates: early endosseous dental implant success and crestal bone changes. A retrospective study. *International Journal of Oral and Maxillofacial Implants*.

[39] Karlsson J., Harmankaya N., Allard S. (2015). Ex vivo alendronate localization at the mesoporous titania implant/bone interface. *Journal of Materials Science: Materials in Medicine*.

[40] Koka S., Babu N. M. S., Norell A. (2010). Survival of dental implants in post-menopausal bisphosphonate users. *Journal of Prosthodontic Research*.

[41] Grant B.-T., Amenedo C., Freeman K., Kraut R. A. (2008). Outcomes of placing dental implants in patients taking oral bisphosphonates: a review of 115 cases. *Journal of Oral and Maxillofacial Surgery*.

[42] Najeeb S., Zafar M. S., Khurshid Z., Zohaib S., Hasan S. M., Khan R. S. (2017). Bisphosphonate releasing dental implant surface coatings and osseointegration: a systematic review. *Journal of Taibah University Medical Sciences*.

[43] Turner A. S. (2001). Animal models of osteoporosis-necessity and limitations. *European Cells and Materials*.

[44] Rosenthal Y., Arami A., Kosashvili Y., Cohen N., Sidon E., Velkes S. (2014). Atypical fractures of the femur related to prolonged treatment with bisphosphonates for osteoporosis. *The Israel Medical Association Journal*.

[45] Hooijmans C. R., IntHout J., Ritskes-Hoitinga M., Rovers M. M. (2014). Meta-analyses of animal studies: an introduction of a valuable instrument to further improve healthcare. *ILAR Journal*.

